# Primary T-cells from human CD4/CCR5-transgenic rats support all early steps of HIV-1 replication including integration, but display impaired viral gene expression

**DOI:** 10.1186/1742-4690-4-53

**Published:** 2007-07-26

**Authors:** Christine Goffinet, Nico Michel, Ina Allespach, Hanna-Mari Tervo, Volker Hermann, Hans-Georg Kräusslich, Warner C Greene, Oliver T Keppler

**Affiliations:** 1 Department of Virology, University of Heidelberg, Heidelberg, Germany; 2 Gladstone Institute of Virology and Immunology, San Francisco, USA; 3 Departments of Medicine and Microbiology and Immunology, University of California San Francisco, San Francisco, USA

## Abstract

**Background:**

*In vivo *studies on HIV-1 pathogenesis and testing of antiviral strategies have been hampered by the lack of an immunocompetent small animal model that is highly susceptible to HIV-1 infection. Since native rodents are non-permissive, we developed transgenic rats that selectively express the HIV-1 receptor complex, hCD4 and hCCR5, on relevant target cells. These animals display a transient low-level plasma viremia after HIV-1_YU-2 _infection, demonstrating HIV-1 susceptibility *in vivo*. However, unlike macrophages, primary CD4 T-cells from double-transgenic animals fail to support viral spread *ex vivo*. To identify quantitative limitations or absolute blocks in this rodent species, we quantitatively assessed the efficiency of key steps in the early phase of the viral replication cycle in a side-by-side comparison in infected cell lines and primary T-cells from hCD4/hCCR5-transgenic rats and human donors.

**Results:**

Levels of virus entry, HIV-1 cDNA synthesis, nuclear import, and integration into the host genome were shown to be remarkably similar in cell lines and, where technically accessible, in primary T-cells from both species. In contrast, a profound impairment at the level of early HIV gene expression was disclosed at the single-cell level in primary rat T-cells and most other rat-derived cells. Macrophages were a notable exception, possibly reflecting the unique transcriptional milieu in this evolutionarily conserved target cell of all lentiviruses. Importantly, transient trans-complementation by *ex vivo *nucleofection with the Tat-interacting protein Cyclin T1 of human origin markedly elevated HIV gene expression in primary rat T-cells.

**Conclusion:**

This is the first study that has quantitatively determined the efficiency of consecutive steps in the HIV-1 replication cycle in infected primary HIV target cells from a candidate transgenic small animal and compared it to human cells. Unlike cells derived from mice or rabbits, rat cells complete all of the early steps in the HIV-1 replication cycle, including provirus integration *in vivo*, with high efficiency. A deficiency in gene expression was disclosed at the single cell level and could be counteracted by the human pTEFb transcription complex factor Cyclin T1. Collectively, these results provide the basis for the advancement of this transgenic rat model through strategies aimed at boosting HIV-1 gene expression in primary rat CD4 T-cells, including human Cyclin T1 transgenesis.

## Background

A highly HIV-permissive rodent with an intact and well-defined immune system would be a boon for the study of HIV pathogenesis and the rapid preclinical evaluation of antiviral strategies. However, native mice and rats cannot be infected by HIV. Species-specific barriers restricting HIV-1 replication in rodents manifest themselves at various steps of the viral replication cycle. Over the past decade, much has been learned about the complex interplay of virus and host, and this work has resulted in a greater molecular understanding of these restrictions in cell lines derived from candidate species, including mice, rats, rabbits, and hamsters. The first important advance was an appreciation of human chemokine receptors, most notably human CCR5 (hCCR5) and CXCR4 (hCXCR4), as cofactors with human CD4 (hCD4) for efficient binding, fusion, and entry of HIV-1 (reviewed in [1]). Indeed, co-expression of hCD4 and hCCR5 or hCXCR4 in cell lines from several small animals [2-9] or in transgenic mice and rats [10-12] is necessary and sufficient for HIV-1 entry, albeit at efficiencies which were suggested to be low [7,9].

More recently, early HIV-1 post-entry barriers have been described in an adherent rabbit cell line [13] and cultured mouse T-cells [5,14], the molecular basis of which has not been defined. Also, efficient Tat-dependent viral gene expression from the HIV-1 long terminal repeat (LTR) occurred in cell lines from mice and hamsters only in the presence of Cyclin T1 of human origin [15]. Orthologues from mouse and hamster, in association with cyclin-dependent kinase CDK9, cannot bind the TAR stem-loop near the 5'-end of nascent HIV-1 transcripts. Efficient HIV-1 transcript elongation by the cellular RNA polymerase II depends on this critical process [16,17]. Interestingly in this context, previous reports suggested considerably higher levels of HIV gene expression in infected, rat-derived Rat2 cells compared to mouse NIH-3T3 and hamster CHO cells, even in the absence of human Cyclin T1 expression [4,7,9].

Additional downstream barriers in rodent cells may limit the production of infectious virus [18]. Specifically, the function of HIV-1 Rev in regulating the splicing and nuclear export of viral transcripts [7,9,19] seems impaired. Moreover, a recessive defect at the level of HIV-1 assembly [7,20] and a maturation or APOBEC3G-dependent infectivity defect [9,21] have been proposed in certain mouse and hamster cell lines, although their severity is still controversial [4,7,9,11].

In contrast, certain rat cell lines co-expressing hCD4 and hCCR5 supported a full HIV-1 replication cycle and the release of infectious virions, although virus production in a single replication cycle was still less than 10% of that in human reference cultures [4]. Thus, the major block to HIV-1 infection in rat cells appeared to be at the level of cellular entry and could be overcome by expression of the HIV-1 receptor complex. Based on these findings, immunocompetent Sprague-Dawley rats were generated that transgenically express hCD4 and hCCR5 selectively on CD4 T-cells, macrophages, and microglia [11]. After systemic challenge with the R5 HIV-1 strain YU-2 (HIV-1_YU-2_), these double-transgenic rats harbored significant levels of episomal HIV-1 cDNA species in lymphatic organs and displayed a low-level plasma viremia up to 7 weeks post-challenge, demonstrating susceptibility to HIV-1 *in vivo *[11]. Furthermore, a recent proof-of-principle study highlighted the utility of these double-transgenic rats for a rapid preclinical evaluation of the inhibitory potency and of the pharmacokinetic properties of antiviral compounds targeting HIV entry or reverse transcription [22].

Although promising, the model still has limitations: levels of plasma viremia were modest and not sustained. This may be due to a cell type-specific block to productive HIV-1 infection in double-transgenic rats. Primary rat macrophages and microglia, but not cultures from T-lymphocytes, could be productively infected by recombinant and primary R5 HIV-1 strains [11]. This barrier to HIV-1 replication in primary rat CD4 T-cells apparently prevented this important cell population from contributing to the viral load *in vivo *and further manipulations of this rodent model may be required to achieve high-level permissivity.

To gain insight into the nature and magnitude of the limitation, the current study focused on a quantitative side-by-side assessment of early steps in the HIV-1 replication cycle in infected primary T-cells from hCD4/hCCR5-transgenic rats and humans. In principle, the exclusive analysis of one HIV cDNA species or gene product would solely reflect a "cumulative" efficiency of all preceding steps and may completely mask severe quantitative deviations, be it higher or lower, in the efficiency of individual steps in the rat-human species comparison. For example, a 10-fold enhancement at the level of HIV entry in hCD4/hCCR5-transgenic rat T-cells could potentially compensate a 10-fold reduction at the level of reverse transcription, resulting in comparable levels of preintegration complexes for subsequent nuclear import and integration. Knowledge on the efficiency of individual steps in the HIV life cycle is thus pertinent for the validation of this HIV-susceptible small animal model and provides the basis for the interpretation and predictive value of *in vivo *infection studies. Consequently, the efficiencies of virion fusion, reverse transcription and nuclear import, provirus formation, and early viral gene expression were analyzed to pinpoint quantitative limitations or absolute blocks in the early phase of replication.

## Results

### Primary T-cells from hCD4/hCCR5-transgenic rats support a productive infection by MoMLV, but not by HIV-1, in ex vivo cultures

**Figure 1:**
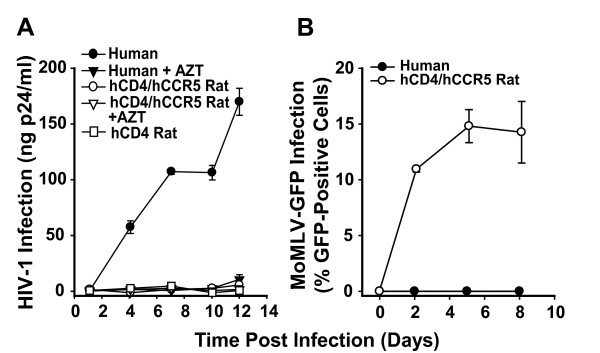
**HIV-1, in contrast to MoMLV, does not spread in primary T-cells from hCD4/hCCR5-transgenic rats**. (A) Activated primary T-lymphocytes from a human donor, a hCD4/hCCR5-transgenic, or a hCD4-single-transgenic rat were infected with HIV-1_YU-2 _(5 ng p24 CA per 2–3 × 10^6 ^cells) overnight and washed. Culture supernatants were monitored for the presence of p24 CA. Where indicated, cultures were treated with AZT (10 μM). (B) The same cultures were exposed to replication-competent ecotropic MoMLV-GFP. Percentages of GFP-positive, productively infected cells were determined by flow cytometry. All values are the arithmetic mean ± S.D. of triplicates. Data are representative for two independent experiments.

We first investigated the ability of HIV-1 to productively infect and spread in primary rat T-cells that transgenically express the HIV-1 receptor complex. Spleen-derived T-cells from a hCD4/hCCR5-transgenic or from a hCD4-transgenic control rat, or T-cells derived from human peripheral blood were infected with HIV-1_YU-2_, and the infection kinetics were followed by monitoring p24 CA concentrations in culture supernatants. As expected, activated human T-cells showed a productive and AZT-sensitive infection (Fig. 1A). In contrast, supernatants from HIV-1_YU-2_-exposed rat T-cell cultures contained only background levels of p24 CA that did not increase over time (Fig. 1A).

To exclude the presence of a broad-spectrum anti-retroviral activity in these rat T-cell cultures, we challenged them in parallel with a replication-competent ecotropic Moloney murine leukemia virus carrying an IRES-*egfp *element in the untranslated region between *env *and the 3'-LTR (MoMLV-GFP). Rat T-cell cultures were highly susceptible to MoMLV-GFP infection, reflected by rapidly increasing percentages of GFP-positive T-cells (Fig. 1B). Conversely, human T-cells did not support a MoMLV-GFP infection, due to the absence of murine cationic amino acid transporter-1, the rodent-specific entry receptor for ecotropic MoMLV [23].

Thus, primary rat T-cells, despite expression of the HIV-1 receptor complex, fail to support a productive and spreading HIV-1 infection, but are highly permissive for infection by a mammalian gamma-retrovirus and thus do not impose a general restriction to retroviral infection.

### HIV-1 efficiently enters primary T-cells from hCD4/hCCR5-transgenic rats

We quantitatively analyzed each early step in the HIV-1 replication cycle with human T-cells serving as a reference. First, the efficiency of HIV-1 entry was assessed in a flow cytometry-based virion-fusion assay [24,25]. T-cells from both species, activated for 5–10 days, were challenged with HIV-1_YU-2 _virions carrying BlaM-Vpr. The cell-permeable CCF2 substrate was introduced into the target cells. After virion fusion, BlaM-Vpr cleaves CCF2, and the altered fluorescence emission serves as a sensitive and specific marker for viral entry. Notably, cell-surface levels of hCD4 were similar, but levels of hCCR5 were markedly higher on CD4 T-cells from transgenic rats than on those from their human counterparts ([11] and data not shown).

**Figure 2:**
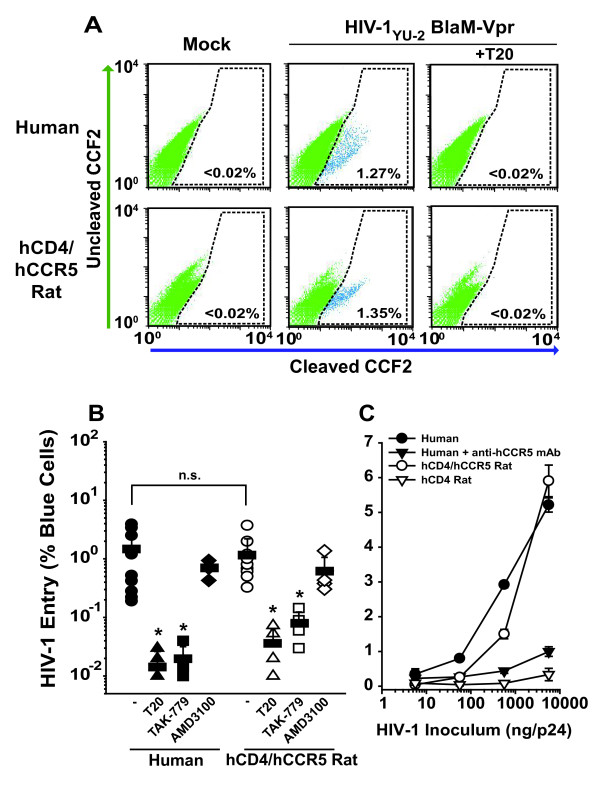
**Transgenic expression of hCD4 and hCCR5 efficiently overcomes the HIV-1 entry block in primary rat T-lymphocytes**. Fusion of HIV-1_YU-2 _virions carrying BlaM-Vpr was analyzed in primary T-cells from humans or hCD4/hCCR5-transgenic rats by multi-parameter flow cytometry [22,25]. (A) Representative FACS dot plots for the detection of cleaved CCF2 substrate, reflecting HIV-1 entry. T-cells from humans (upper panels) and double-transgenic rats (lower panels) were either mock-infected (left panels) or infected with HIV-1_YU-2 _(50 ng HIV-1 p24 CA per 2–3 × 10^6 ^cells), either without (middle panels) or with (right panels) the fusion inhibitor T20 (50 μM). (B) Results from virion-fusion assays with T-cells from 5–9 different donors per species. Were indicated, the CCR5 antagonist TAK-779, the CXCR4 antagonist AMD3100 (both 1 μM), or T20 (50 μM) were added 15–30 min before virus challenge. Symbols indicate arithmetic means of triplicates from one virion-fusion experiment; horizontal bars depict the arithmetic mean ± S.E.M. of all experiments (n.s. = not significant; p = 0.66; * p ≤ 0.02) (C) Titration of HIV-1_R7/3_YU-2 Env GFP carrying BlaM-Vpr in virion-fusion assays on primary T-cells from both species. Where indicated (filled triangle) the anti-hCCR5 mAb 2D7 (50 μg/ml) were added to cells 15–30 min before virus challenge.

The percentages of T-cells from hCD4/hCCR5-transgenic rats and humans that allowed HIV-1_YU-2 _entry was statistically indistinguishable (1.2 ± 1.0% and 1.4 ± 1.3%, respectively; p = 0.66; n.s.; Mann-Whitney *U *test) (Fig. 2A and 2B). Mock-infected T-cells or T-cells that had been treated either with the fusion inhibitor enfuvirtide (T20) or with the CCR5 antagonist TAK-779 before exposure to the cell-free viral inoculum (50 ng p24 CA) displayed only background levels of cleaved CCF2-positive cells (Fig. 2A and 2B). As an additional control of specificity, the CXCR4 antagonist AMD3100 did not significantly (p = 0.9 (human); p = 0.2 (rat)) affect the ability of the R5 HIV-1 strain to fuse with these primary T-cells.

To explore species-specific differences in the relationship between the dose of inoculum and the efficiency of virion fusion, primary T-cells were exposed to increasing doses of HIV-1_R7/3_YU-2 Env GFP carrying BlaM-Vpr (Fig. 2C, the presence of the GFP gene is unimportant in this assay). In a titration of the inoculum covering three orders of magnitude, T-cells from hCD4/hCCR5-transgenic rats supported HIV-1 entry at levels that closely matched those of their human counterparts (Fig. 2C). Human T-cells, which had been pretreated with an anti-CCR5 antibody, and T-cells from a hCD4-transgenic rat served as controls and were largely refractory to virion fusion. In summary, these results show that expression of the HIV-1 receptor complex on primary rat T-cells efficiently overcomes the HIV-1 entry block. This suggests that limitations further downstream in the replication cycle restrict productive infection in these rodent cells.

### Nuclear import of de novo synthesized viral DNA genomes is similar in primary T-cells from rats and humans

Next, we determined if the HIV-1 replication defect in primary rat T-cells could be accounted for by a reduced efficiency of reverse transcription or nuclear import of newly synthesized HIV-1 cDNA, as suggested for mouse T-cells [5,14]. To ensure comparable conditions in the cross-species comparisons, infections were genetically limited to a single round. As a consequence, the absolute levels of individual processes in this primary cell type were generally low. Infections were conducted with HIV-1 generated from a replication-deficient HIV-1_NL4-3_E^- ^GFP backbone pseudotyped with YU-2 Env. This approach allowed a kinetic analysis of the formation of HIV-1 2-LTR circles. 2-LTR circles are an episomal HIV cDNA species, are formed exclusively in the nucleus by cellular ligases of the non-homologous DNA end joining pathway [26], and serve as a quantitative marker for reverse transcription and nuclear import of the viral cDNA genome [27].

**Figure 3:**
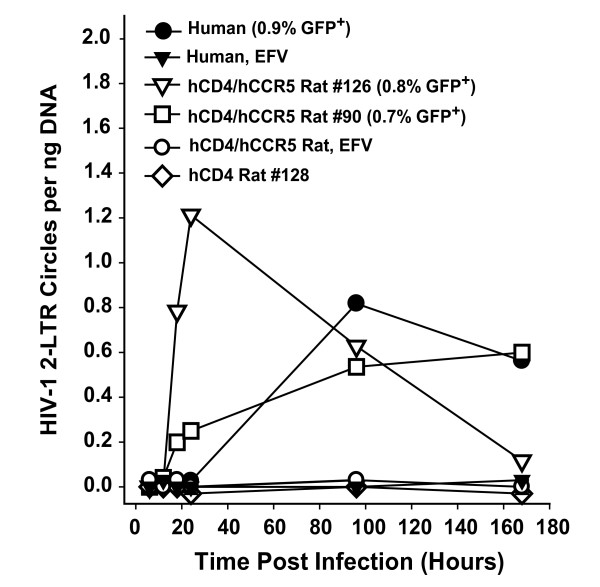
**Reverse transcription and nuclear import of *de novo *synthesized HIV-1 cDNA are well supported in T-cells from hCD4/hCCR5-transgenic rats**. Primary T-cells from a human donor or from transgenic rats were exposed to YU-2 Env pseudotyped HIV-1_NL4-3_E^- ^GFP. At the indicated time points, post-infection samples were taken from cultures, and the relative levels of 2-LTR circles in cell extracts were scored by quantitative PCR. The percentage of GFP-positive cells at day 4 after infection is given in parentheses.

2-LTR circles were detected in infected primary T-cells from both species, and peak levels differed by no more than twofold (Fig. 3). In contrast, no 2-LTR circles could be detected in efavirenz-treated cultures or cultures from a hCD4-single-transgenic rat, demonstrating that the amplified episomal HIV-1 cDNAs had been generated *de novo *after a receptor-complex-mediated infection and were not present in the inoculum. Furthermore, flow cytometric analysis 96 h after infection showed similar percentages of T-cells expressing GFP from the *nef *locus (0.7–0.9% GFP-positive cells, Fig. 3) for infected cultures from both species, and DNA extracts from samples taken at the same time point contained comparable levels of 2-LTR circles (0.53–0.81 copies per ng of DNA; Fig. 3). Thus, infected primary rat T-cells appear to support reverse transcription and nuclear import of *de novo *synthesized HIV-1 cDNA at levels similar to human reference cells, and early HIV gene products can be expressed. These results suggest that limitations underlying the replication block in infected T-cells from this rodent species must be acting at a step after nuclear entry of the preintegration complex.

### A quantitative nested PCR to detect integrated HIV-1 DNA in rat cells

To assess the next major step in the HIV-1 replication cycle, we quantified provirus formation in infected rat cells. In principle, a defect at the level of integration can completely abrogate HIV-1 replication, but may still allow expression of early viral proteins, including Nef, from episomes in the first round of infection [28,29].

**Figure 4:**
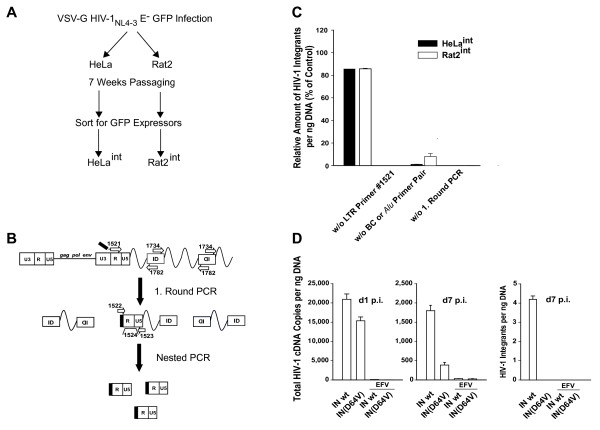
**Establishment and validation of a real-time PCR for HIV-1 integrants in rat cells**. (A) Schematic of the generation of Rat2^int ^(rat) and HeLa^int ^(human) cells, carrying HIV-1_NL4-3_E^- ^GFP, as species-specific HIV-1 integration standards. (B) PCR strategy of the nested rat integration PCR. In the first round of PCR, a segment of integrated HIV-1 cDNA was amplified by one primer annealing in the HIV-1 LTR (primer #1521) and two outward-facing primers targeting the rat ID element (primers #1734 and #1782). To increase specificity, LTR primer #1521 contains a lambda-phage heel sequence at the 5'-end [30]. In a nested, second-round PCR, a lambda-specific primer (primer #1522), a second LTR primer (primer #1523), as well as an HIV-1 LTR-specific probe (probe #1524) were employed to exclusively amplify products generated during the first-round PCR. (C) Technical validation of species-specific integration PCR on Rat2^int ^or HeLa^int ^[30] cells. Levels of HIV-1 integrants from the complete standard PCR reaction were arbitrarily set to 100%, and levels determined for several specificity controls (omission (w/o) of LTR primer #1521, omission (w/o) of cellular anchor primer pair (BC, #1734 and #1782 (rat) or *Alu*, #1519 and #1520 (human)), omission (w/o) of first-round PCR reaction) are given relative to that. (D) Validation of rat integration PCR. Parental Rat2 cells were infected with VSV-G pseudotyped HIV-1 GFP vectors carrying either a wildtype integrase (IN wt) or catalytically inactive integrase (IN(D64V)). Where indicated, efavirenz (5 μM) was added 1 h before infection. Cultures of infected Rat2 cells were monitored for the presence of total HIV-1 cDNA on day 1 (left panel) or day 7 (middle panel) post infection. On day 7, cells were also analyzed for the presence of integrated HIV-1 cDNA (right panel).

Similar to a reported nested PCR strategy to specifically amplify HIV-1 integrated in proximity to genomic *Alu *repeat elements in human cells [30], we designed a nested real-time PCR assay to detect integrated HIV-1 provirus in rat cells by employing an ID consensus sequence within the rat BC1 RNA gene [31,32] as the rodent repeat target for the cellular anchor primer pair. To serve as standards for species-specific quantitative analyses of provirus formation, stable populations of human and rat cell lines containing integrated HIV-1 proviruses were generated (Fig. 4A): adherent HeLa (human) and Rat2 (rat) cells were infected with VSV-G pseudotyped HIV-1_NL4-3_E^- ^GFP at a low multiplicity of infection and subsequently passaged for 7 weeks to allow complete loss of unintegrated HIV-1 cDNA species. After an overnight-stimulation with the histone deacetylase inhibitor trichostatin A, GFP-expressing cells were enriched by flow cytometric sorting, and bulk cultures of these provirus-containing, heterogeneous cell populations, named HeLa^int ^and Rat2^int^, were expanded. Since these cells no longer contain unintegrated HIV-1 cDNA species, the absolute number of integrated proviruses per ng cellular DNA in HeLa^int ^and Rat2^int ^could be accurately determined by quantifying the absolute number of HIV-1 cDNA by real-time PCR [22], thus providing an integration standard. These values were 6.3 and 5 HIV-1 integrants per ng DNA for Rat2^int ^and HeLa^int^, respectively.

The PCR strategy for the newly developed integrated provirus in rat cells is depicted in Fig. 4B and described in detail in the figure legend. This rat integration PCR and a human integration PCR, the latter essentially following a published protocol [30], were validated side-by-side using genomic DNA from Rat2^int ^or HeLa^int ^cells, respectively (Fig. 4C). The numbers of HIV-1 integrants per ng DNA were set to 100%. First, omission of LTR primer #1521 from the first-round reaction resulted in a loss of the amplification signal. Second, a reaction mix without the cellular primer pair (#1734 and #1782 (rat); #1519 and #1520 (human)) yielded low signals (9.2% for Rat2^int ^and 1.3% for HeLa^int^), most likely due to the partial formation of single-stranded DNA from LTR-containing HIV-1 cDNA by the first-round LTR primer, as previously suggested [30]. Finally, omission of the first-round PCR reaction yielded no signal above background, indicating that second-round amplification of non-preamplified DNA is not a disturbing factor (Fig. 4C).

As an additional validation of the rat integration PCR, we quantified levels of total HIV-1 cDNA and integrated HIV-1 cDNA in parental Rat2 cells infected with either an integration-competent or an integration-defective lentiviral vector, the latter carrying the IN(D64V) catalytic core mutation [33]. On day 1 after infection, high levels of total HIV-1 cDNA, which were not detectable after efavirenz pre-treatment of cells, were amplified from Rat2 cells challenged with either lentiviral vector (Fig. 4D, left panel). In cell extracts obtained on day 7 after infection, levels of total HIV-1 cDNA had decreased to 2.8–8.6% of the levels on day 1. Most importantly, while integrants were readily amplified by the newly developed PCR strategy at this late time point in Rat2 cells infected with the IN wt vector, provirus formation could not be detected in cells infected with the IN(D64V) vector (Fig. 4D, right panel). In summary, we have established and validated a real-time PCR for the quantitative detection of HIV-1 integrants in infected rat cells.

### HIV-1 integrates into the genome of rat cells, infected in vitro or in vivo, as efficiently as into the genome of human cells

**Figure 5:**
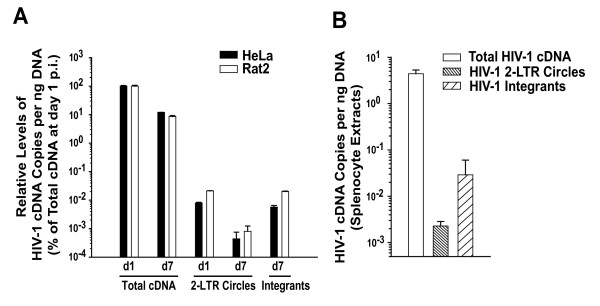
**Integration of HIV-1 into the genome occurs efficiently in infected rat cells**. (A) Parental Rat2 cells and HeLa cells were exposed to VSV-G pseudotyped HIV-1 GFP vectors and cultivated for 7 days. The relative levels of total HIV-1 cDNA, 2-LTR circles, and integrants were quantified by specific real time PCR in extracts from cell aliquots taken at the indicated time points. All copy numbers per ng DNA are depicted relative to the levels of total HIV-1 cDNA on day 1 after infection, levels of which were arbitrarily set to 100%. (B) Three hCD4/hCCR5-transgenic rats and one hCCR5-single-transgenic rat were challenged intravenously with HIV-1_YU-2_. On day 4, all animals were sacrificed and spleens removed. The levels of all three HIV-1 cDNA species were quantified in splenocytes extracts relative to a rat GAPDH standard by real-time PCR. Results are presented as the arithmetic mean ± S.E.M. of data obtained for the three double-transgenic rats.

To assess the kinetics of formation of different HIV-1 cDNA species and the integration frequency in infected rat cells, parental Rat2 cells and HeLa cells were simultaneously challenged with a VSV-G pseudotyped lentiviral vector. DNA extracts of cell aliquots taken from infected cultures at days 1 and 7 after infection were analyzed for levels of total HIV-1 cDNA and 2-LTR circles [22], as well as integrants by the assay described above (Fig. 5A). As a normalisation reference, the level of total HIV-1 cDNA obtained for each cell line at day 1 after infection was set to 100%.

Notably, the relative levels of total HIV-1 cDNA and of 2-LTR circles at days 1 and 7 after infection were similar in infected Rat2 and HeLa cells. In both species, the latter episomal DNA species accounted for ~0.01% (day 1) and ~0.001% (day 7) of total HIV-1 cDNA. The 90% reduction likely reflects the gradual loss of episomes through cell divisions. At this late time point, the relative levels of integrants in infected Rat2 and HeLa cells were again quite similar and represented ~0.02% or ~0.005% of the total HIV-1 cDNA at day 1, respectively. Together, the relative abundance of these three HIV-1 cDNA species was remarkably similar in these infected cultures of adherent cells of rat and human origin. Unfortunately, reliable detection of HIV-1 integrants in cultured primary T-cells was precluded by a virus stock production-related contamination with proviral plasmid DNA that was partially resistant to DNAse treatment (data not shown).

In a recent infection study in hCD4/hCCR5-transgenic rats, we observed that this plasmid contamination of virus stocks is apparently lost or degraded *in vivo*, allowing the exclusive detection of HIV-1 cDNAs synthesized *de novo *in splenocyte extracts [22]. Consequently, we challenged three hCD4/hCCR5-transgenic rats and one hCCR5-single-transgenic control rat intravenously with HIV-1_YU-2_. Four days after infection, animals were sacrificed, and the spleens, which harbor high levels of CD4 T-lymphocytes, were removed. Levels of total HIV-1 cDNA in splenocyte extracts from infected hCD4/hCCR5-transgenic rats ranged from 3.2 to 5.3 copies per ng DNA (Fig. 5B). As important controls of specificity, neither HIV-1 cDNA nor HIV-1 integrants could be amplified from extracts of the hCCR5-transgenic animal challenged with the identical virus inoculum (data not shown). Furthermore, no HIV-1 integrants could be amplified from splenocyte DNA derived from efavirenz-treated, infected hCD4/hCCR5-transgenic rats ([22] and data not shown). Collectively, these results demonstrate that the signals obtained from samples derived from double-transgenic rats indeed represent *de novo*-synthesized HIV-1 cDNA. Here, integrants were detected at a frequency of 0.03 ± 0.02 copies per ng DNA, representing 0.65% of the total HIV-1 cDNA at this time point after infection. Relative levels of 2-LTR circles were clearly less abundant (0.0023 ± 0.0006 copies per ng of DNA), representing 0.05% of the total HIV-1 cDNA.

Thus, HIV-1 integrants can be quantitatively detected in splenocyte extracts from hCD4/hCCR5-transgenic rats following *in vivo *challenge, and the relative representation of the three HIV-1 cDNA species analyzed mirrors the results obtained for *in vitro *infection studies in cell lines from both species (Fig. 5A). This suggests that the integration frequency into the genome of rat T-lymphocytes is not impaired.

### Single-cell analysis reveals that early HIV-1 gene expression is diminished in infected primary rat T-cells

Subsequently, we sought to compare levels of early HIV gene expression in primary T-cells on a single cell level. Activated T-cell cultures from hCD4/hCCR5-transgenic rats and human donors were infected with single-round HIV-1_NL4-3_E^- ^GFP viruses pseudotyped with YU-2 Env and analyzed for the expression of the GFP reporter, at the *nef *locus, by flow cytometry.

**Figure 6:**
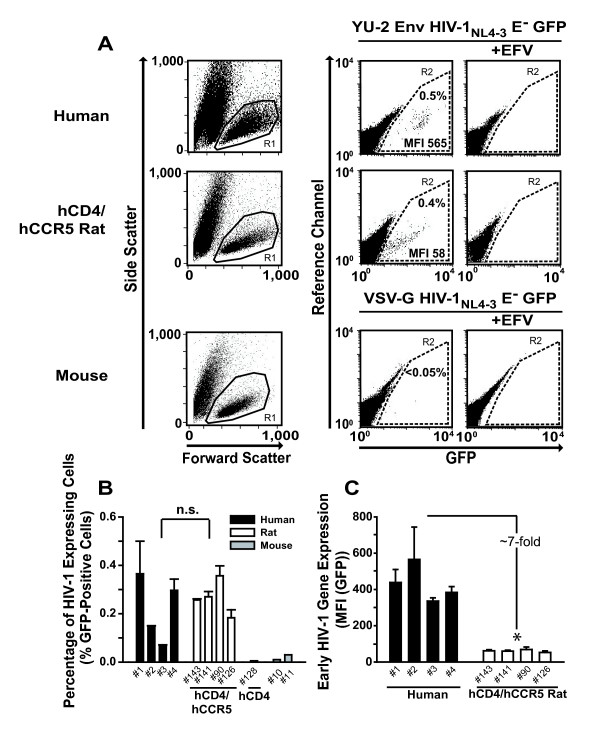
**Activated primary rat T-cells exhibit a profound block to HIV-1 replication at the level of early HIV-1 gene expression**. (A) Representative FACS dot plots of T-cells from a human donor, a hCD4/hCCR5-transgenic rat, and a BALB/c mouse infected with the indicated HIV-1 GFP reporter viruses (50 ng p24 CA per 2–3 × 10^6 ^cells) and analyzed for GFP expression on day 6 after infection. Viable cells were identified by gating on the live lymphocyte population (gate R1) in the FSC/SSC plot (left vertical panels). Gate R2 defines the GFP-positive subpopulation of gate R1. Shown are results obtained from cells infected in the absence (middle vertical panels) and presence of efavirenz (EFV) (right vertical panels). (B) Percentage of GFP-positive cells obtained for T-cell cultures from four human donors, four hCD4/hCCR5-transgenic rats, and two BALB/c mice which had been infected as described in A (p = 0.77; n.s.) (C) MFI(GFP) of infected human and rat T-cell cultures shown in B (p = 0.02; * = significant).

**Figure 7:**
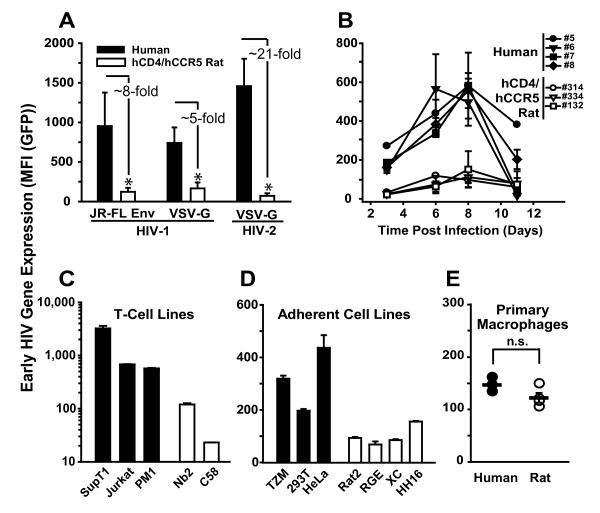
**Early HIV-1 gene expression is also diminished in infected rat cell lines, but not in infected primary rat macrophages**. (A) T-cell cultures were challenged with HIV-1_NL4-3_E^- ^GFP pseudotyped with either JR-FL Env (n = 2) or VSV-G (human (n = 12); rat (n = 24)), or challenged with VSV-G pseudotyped HIV-2_ROD-A_E^- ^GFP (human (n = 7); rat (n = 12)) and analyzed for GFP expression at day 3 after infection as described in Fig. 6. Results represent the arithmetic mean ± S.E.M. of the indicated number of independent T-cell cultures (p = 0.009; * = significant). (B) Kinetics of GFP expression in primary T-cell cultures from human donors or hCD4/hCCR5-transgenic rats infected with single-round YU-2 Env pseudotyped HIV-1_NL4-3_E^- ^GFP. The percentage of GFP-positive cells was quantified every 2–3 days by flow cytometry. Results are the arithmetic mean ± S.D. from individual T-cell cultures. (C) Human T-cell lines SupT1, Jurkat, or PM-1, and rat T-cell lines Nb2 or C58, as well as (D) human adherent cell lines TZM, 293T, or HeLa, and rat adherent cell lines Rat2, RGE, XC, or HH16, and (E) primary macrophages from both species (human (n = 3); rat (n = 4)) were exposed to VSV-G pseudotyped HIV-1_NL4-3_E^- ^GFP at a low MOI to achieve single cell infections. The MFI of GFP expression was determined on day 3 after infection (p = 0.2; n.s.). Results shown in (C-D) represent the arithmetic mean ± S.D. of triplicates. Circles in (E) indicate the arithmetic mean of triplicates from one experiment and the horizontal bar shows the arithmetic mean ± S.E.M. of all donors/animals analyzed.

The percentages of GFP-expressing T-cells 3 days after infection were comparable and efavirenz-sensitive in both species (Fig. 6A (gate R2)), and only donor-specific variations were noted (Fig. 6B). Most remarkably, however, the intensity of GFP expression, reflected by the mean fluorescence intensity (MFI) of individual infected cells analyzed, drastically differed: infected human T-cells displayed a rather distinct population of GFP high-expressing cells (Fig. 6A (upper horizontal panel); quantification in Fig. 6C), whereas T-cells from hCD4/hCCR5-transgenic rats exhibited only rather low levels of expression of the early gene product (Fig. 6A, middle horizontal panel; Fig. 6C). On average, this difference in gene expression was 5- to 8- fold, irrespective of the viral entry route (HIV-1_YU-2 _Env, HIV-1_JR-FL _Env, VSV-G) (Fig. 6A,C; Fig. 7A). Interestingly, this species-dependent gap was even more pronounced for an HIV-2_ROD-A_E^- ^GFP reporter virus, which showed a 21-fold difference (Fig. 7B). Remarkably, primary T-cells from native BALB/c mice were largely refractory to infection by VSV-G pseudotyped HIV-1_NL4-3_E^- ^GFP (Fig. 6A, lower horizontal panel, and 6B), and virtually no GFP-positive cells could be detected despite efficient virion entry (data not shown), consistent with previous reports [5,14].

We next asked whether this striking difference in gene expression levels between primary human and rat T-cells could be due to species-specific differences in the kinetics of HIV-1 gene expression. Monitoring GFP expression over the course of 11 days (Fig. 7B), rat T-cells infected with the replication-deficient HIV-1 reporter virus did not reveal significant alterations in their levels of early gene expression. GFP expression levels in human reference cultures peaked at days 6–8 after infection and subsequently decreased, most likely due to gene silencing or loss of infected cells from the culture. Thus, a mere delay in viral gene expression in infected rat T-cells seems unlikely.

Furthermore, this phenotype turned out to be largely species-specific: the defect in early HIV-1 gene expression was seen most drastically in infected T-cell lines from rats, with a factor of difference ranging from 6- to 100-fold (Fig. 7C), and also in adherent cell lines, albeit less pronounced (4- to 10-fold factor of difference, Fig. 7D). Interestingly, primary macrophages were an exception, revealing comparable levels of early HIV-1 gene expression in both species after infection with the VSV-G pseudotyped HIV-1_NL4-3_E^- ^GFP reporter virus (Fig. 7E; p = 0.2; n.s.).

Collectively, these flow cytometric data at single cell level demonstrate a major post-integrational limitation in viral gene expression in most rat-derived cells, which may be a key reason for the failure of primary T-cells from hCD4/hCCR5-transgenic rats to support HIV-1 replication.

### Transient expression of human Cyclin T1 in rat T-cells boosts early HIV gene expression

**Figure 8:**
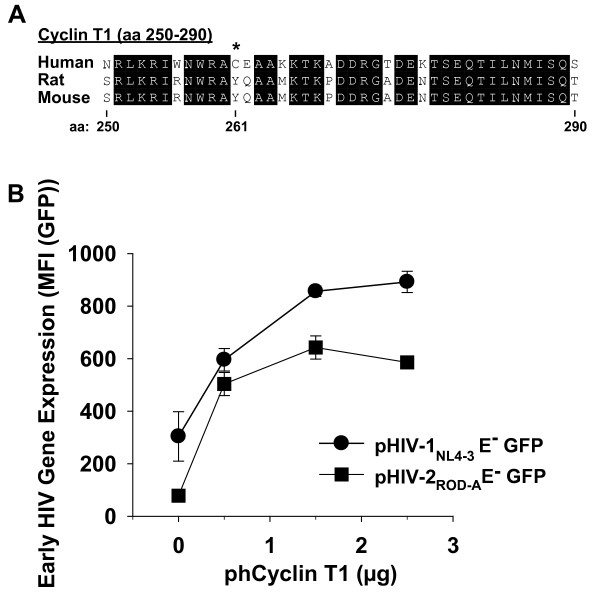
**Transient expression of human Cyclin T1 in primary rat T-cells boosts early HIV gene expression**. (A) Sequence alignment of amino acids 250–290 of human, rat and mouse Cyclin T1. * depicts the critical amino acid position 261. (B) Proviral pHIV-1_NL4-3 _GFP or pHIV-2_ROD-A _GFP constructs were co-transfected with increasing amounts of an expression vector encoding for either human Cyclin T1 (phCyclin T1) or no transgene (pcDNA3.1), and cells were analyzed for GFP expression 20 h later by flow cytometry.

On a molecular level, the inability of mouse Cyclin T1 to support the Tat-mediated enhancement of HIV transcription has been mapped to the C261Y variation in the mouse protein [34] (parts of the amino acid sequence are shown in Fig. 8A). Intriguingly, rat Cyclin T1 (genebank: Ccnt1_predicted XM_235633) shares around 96% sequence homology with the mouse orthologue, including the critical C261Y variation ([4]; Fig. 8A).

To rapidly assess whether expression of human Cyclin T1 can affect HIV gene expression in primary rat T-cells we performed transient co-transfection studies employing a recently developed species-adapted non-viral gene delivery method based on the nucleofection technology [35]. Proviral pHIV-1_NL4-3 _GFP or pHIV-2_ROD-A _GFP constructs were co-transfected with increasing amounts of an expression vector encoding for either human Cyclin T1 or no transgene, and cells were analyzed for GFP expression 20 h later by flow cytometry. Remarkably, expression of human Cyclin T1 enhanced early HIV gene expression in a concentration-dependent and saturable manner in these primary rodent T-cells (Fig. 8B). As a control of specificity, human Cyclin T1 expression did not affect reporter gene expression from a CMV immediate early promoter-driven construct, pEGFP-N1, in nucleofected rat T-cells (data not shown). Moreover, co-transfection of phCyclin T1 and these proviral constructs into primary T-cells from human donors did not significantly alter levels of HIV gene expression (data not shown), indicating that physiological levels of Cyclin T1 are not limiting in these cells.

Collectively, these transient *ex vivo *studies suggest an underlying transcriptional defect linked to the non-functional rat orthologue and demonstrate a beneficial effect of Cyclin T1 of human origin on HIV gene expression in primary rat T-cells.

## Discussion

An in-depth assessment of the individual steps in the viral replication cycle in primary target cells is critical for generating a highly HIV-1-permissive immunocompetent rodent model for HIV-1 infection. Here we used quantitative assays to probe consecutive steps in the early phase of HIV-1 infection. We found that virion fusion, reverse transcription, nuclear import, and HIV-1 integration into the host genome occur with similar efficiencies in primary T-cells from hCD4/hCCR5-transgenic rats and humans. However, one step downstream in the replication cycle, we find viral gene expression to be greatly diminished in infected T-cells from rats, ~sixfold for HIV-1_NL4-3 _and ~20-fold for HIV-2_ROD-A _reporter viruses, relative to human references, identifying the next relevant replication barrier to be tackled in this species. Furthermore, we show a beneficial effect of Cyclin T1 of human origin on HIV gene expression in primary rat T-cells, providing a strong rationale for the generation of a novel human Cyclin T1-transgenic rat line.

We began our examination with the entry of the virus into the cell. Expression of hCD4 and a major human chemokine coreceptor is required for entry into cells of non-human origin, but the efficiency of virus entry into rodent cell lines expressing high levels of the HIV-1 receptor/coreceptor complex was thought to be very low (0.25–17% of human cells) [9]. To directly compare the efficiencies of R5 HIV-1 entry in T-cells from hCD4/hCCR5-transgenic rats and human reference cultures, we employed a sensitive and specific virion-fusion assay [22,24,25]. The HIV-1 receptor/coreceptor complex at the expressed surface levels is fully functional in rats, implying correct post-translational modifications, receptor trafficking, and cell-surface topology of both receptor components. Moreover, our results provide the first quantitative demonstration of the success of entry receptor transgenesis towards the development of an HIV-1-permissive rodent model.

Next we assessed the efficiency and kinetics of HIV-1 cDNA synthesis, nuclear import of the preintegration complex (2-LTR circle formation), and provirus integration by real-time PCR. Our post-entry analyses in primary T-cells and a fibroblast cell line from rats suggest an efficient progression of the viral replication cycle from reverse transcription over nuclear import to integration. For primary T-cells, technical limitations did not allow us to conduct a direct rat-human species comparison for HIV cDNA synthesis. However, comparable levels of HIV entry and 2-LTR circle formation, representing the preceding and subsequent step in the replication cycle, strongly suggest that reverse transcription occurs efficiently in primary rat T-cells, although subtle quantitative differences cannot be excluded.

These observations argue that rat-derived cells are intrinsically more permissive for these early post-entry steps in HIV-1 infection than cells from other small animals, although this difference is not understood at a molecular level. In this context, two other studies found that defects in reverse transcription and/or nuclear import in infected mouse T-cells severely impede viral gene expression and appear to be recessive in nature [5,14]. Furthermore, aberrant intracytoplasmic trafficking of the reverse transcription complex in rabbit SIRC cells may be an underlying reason for a reduced HIV-1 cDNA synthesis in this rodent-like species [13].

Contrasting the intact HIV-1 entry and early post-entry steps, we identified a limitation at the level of early HIV-1 gene expression in primary T-cells from double-transgenic rats that was independent of the viral entry route and that was apparent in all T-cell lines and adherent cell lines analyzed. We used flow cytometry to quantify early HIV-1 gene expression through the MFI of GFP as a surrogate for Nef. This approach allowed a resolution at a single-cell level in infected cultures rather than bulk analyses using luciferase or chloramphenicol-acetyl-transferase reporter systems applied in earlier studies [4,9,11,36]. Our experimental strategy was particularly useful for the cross-species comparison since the analysis of gene expression is much less affected by differences in the efficiency of preceding steps in the replication cycle. Specifically, earlier studies assessed HIV-1 gene expression in rodent cell lines after infection with VSV-G HIV-1 luciferase reporter viruses [4,7,9], based on the assumption that the efficiency of VSV-G-mediated entry is comparable across species. However, in quantitative virion-fusion assays, we found generally greater VSV-G susceptibility in rat cells than human references, ranging from 3- to 10-fold for an identical inoculum in adherent cell lines (Rat2 versus HeLa), as well as in primary T-cells and macrophages (data not shown). This previously unrecognized difference may have led to an overestimation of the capacity of Rat2 cells to support HIV-1 gene expression [4,7,9]. Moreover, alternative transcriptional assays based on the transient transfection of LTR reporter and Tat expression constructs rely on a normalisation of transfection efficiency that involves a third, typically CMV immediate early promoter-driven reporter, for which species- and cell type-specific differences in the activity may be an additional confounding problem.

We reasoned that an impaired activity of the Tat-dependent HIV-1 LTR transactivation [16,17] may underlie the inefficient early gene expression for HIV-1_NL4-3 _and HIV-2_ROD-A _reporter viruses in primary rat T-cells. In mice, the inability of Cyclin T1 to support the efficient interaction with the TAR element when bound to Tat was functionally mapped to one essential amino acid (C261Y) [18,34,37,38], and intriguingly, rat Cyclin T1 and the mouse orthologue both have a tyrosine at this position [4]. Here, using recently developed technology [35], we could demonstrate a marked enhancement of HIV gene expression in primary rat T-cells following transient expression of human Cyclin T1. This suggests that the impaired HIV gene expression, at least in part, is due to a transcriptional defect linked to a non-functional rat Cyclin T1. Furthermore, these *ex vivo *studies provide a sound rationale for the generation of human Cyclin T1-transgenic rats as an additional genetic modification of the hCD4/hCCR5-transgenic rat model of HIV infection.

In addition, and despite the fact that the frequency of HIV-1 integration into the rat genome appeared to be normal, an underlying defect in the HIV-1 integration site selection must be considered. The efficiency of viral transcription is governed by characteristics of the chromatin environment at the integration site [39]. Whereas HIV-1 preferentially integrates within active transcription units [40], MLV favours integration at transcription start regions [41,42]. The dependence of this process on host factors, such as lense epithelium-derived growth factor (LEDGF/p75), that is required for HIV-1, but not for MLV integration [43,44], makes this a candidate step for species-specific disturbances of integration site selection.

As an interesting observation, infected macrophages pose an exception to the otherwise species-specific impairment at the level of early HIV-1 gene expression in the rat-human species comparison. This is particularly noteworthy since macrophages from hCD4/hCCR5-transgenic rats are the only rodent cells known to support a productive and spreading HIV-1 infection, albeit at lower levels than in human monocyte-derived macrophages [4,11]. Providing an intriguing explanation, HIV-1, in part, exploits a distinct set of nuclear transcription factors and alternative mechanisms of transcriptional regulation in macrophages than in other cell types, including T-cells (reviewed by [45-47]). From the perspective of viral evolution, it is remarkable that macrophages are the only primary cell type that is permissive for all lentiviruses in their respective host [48]. Their marked phenotypic difference in the relative ability to support early gene expression of HIV-1_NL4-3 _and HIV-2_ROD-A _reporter viruses compared to T-cells may reflect their higher dependence on specific transcription factors, including NF-kB and NFAT-1.

Pursuing a conceptually different approach to the generation of an HIV small animal model, several HIV xenotransplant models have been developed by introducing human hematopoietic cells or foetal tissues into immunodeficient strains of mice. Such animals support local HIV replication in grafts or, following recent advancements, also systemic HIV replication with high level viremia (reviewed by [49]). Unfortunately, adaptive immune responses in such "humanized" mice were reported to be low or absent, and these technically challenging models are not amenable to predictive high-throughput screenings of drug candidates.

## Conclusion

Collectively, the efficiency of all early steps of the HIV-1 replication cycle in CD4 T-cells from hCD4/hCCR5-transgenic rats, in clear contrast to mice, up to and including provirus formation is high. These results are encouraging for the block-by-block development of a fully permissive rat model of HIV-1 disease. From a different perspective, these results provide an important validation of these HIV-susceptible small animals by underscoring the predictive value of hCD4/hCCR5-transgenic rats as a model for the evaluation of antiviral compounds targeting early events of HIV-1 replication [22]. Furthermore, the demonstration of a marked quantitative limitation at the level of viral gene expression in rat CD4 T-cells and, importantly, the beneficial effect of human Cyclin T1 in these primary cells, provide the basis for the design of strategies to overcome it.

## Methods

### Animals

Generation and initial characterization of hCD4/hCCR5-transgenic rats on an outbred Sprague-Dawley background have been reported [11]. Female BALB/c mice were obtained from Charles River Laboratories (Sulzfeld, Germany). Animals were housed in the central animal facility of the University of Heidelberg. Animal experiments were conducted according to the German animal welfare act and with authorization of the Regierungspräsidium Karlsruhe (35-9185.81/G-100/02 and 34395) and supervised by animal welfare officers of the University of Heidelberg.

### Cells

Cultures of primary T-cells and macrophages from randomly selected human donors and transgenic rats and BALB/c mice were generated, activated, and cultivated as reported [11,22,35,50]. Rat cell lines Rat2 (rat fibroblast-like cell line [ATCC CRL-1764]), RGE (rat glomerular endothelial cell line [DSMZ ACC 262]), XC (rat sarcoma cell line [DSMZ ACC 118]), HH16.cl.4 (rat mammary tumor cell line [DSMZ ACC 358]), C58 (rat T-cell lymphoma [ATCC TIB-236]), and Nb2 (rat T-cell lymphoma) [11] were cultivated as recommended by the original sources. Human cell lines SupT1, Jurkat, HeLa, PM-1, TZM-bl and 293T were cultivated as reported [4,50].

### Virus stocks

Generation of replication-competent HIV-1_YU-2 _and HIV-1_R7/3_YU-2 Env GFP stocks has been reported [11]. HIV-1_YU-2 _virions carrying β-lactamase-Vpr chimeric fusion proteins (BlaM-Vpr) were produced by triple-transfection of 293T cells as described [24] with pYU-2 proviral DNA (60 μg), pBlaM-Vpr (20 μg), and pAdVantage (8 μg) vectors (Promega, Madison, WI) per 15-cm^2 ^dish by calcium phosphate DNA precipitation. The molecular clone pNL4-3 E^- ^GFP, carrying an *egfp *gene within the *nef *locus driven by the 5'- LTR, was a kind gift of Dr. Nathaniel Landau (New York University, New York, NY) [51] via the NIH AIDS Research and Reference Reagent Program. Pseudotyping with VSV-G, JR-FL Env and YU-2 Env was performed as reported [4]. All HIV-1 stocks were characterized for p24 CA concentration by antigen enzyme-linked immunosorbent assay and/or for infectious titer on TZM-bl cells [50]. The molecular clone pHIV-2_ROD-A _was a gift from Dr. Matthias Dittmar (Department of Virology, University of Heidelberg, Germany) [52]. Lentiviral vectors were generated by triple-transfection of 293T with pΔR8.91, pHR.GFP [53] and pVSV-G by calcium phosphate DNA precipitation and were DNAse-treated (Turbo DNAse, Ambion, Dresden, Germany) for 1 h at 37°C (1 unit DNAse/10 μl of concentrated virus stock). MoMLV-GFP was constructed by introducing the *egfp *gene driven from an internal ribosomal entry site (IRES) into the untranslated region between the *env *gene and the 3'-LTR of MoMLV at unique *Not*I/*Mlu*I sites. Titers and replication kinetics of MoMLV-GFP and MoMLV wildtype were similar, and GFP expression of the recombinant was stable over several passages on mouse fibroblasts (data not shown).

### HIV-1 virion-fusion assay

The HIV-1 virion-fusion assay based on flow cytometry was conducted essentially as described [22,24,25]. Briefly, primary T-cells were pretreated with the indicated concentrations of enfuvirtide (Roche, Indianapolis, IN), TAK-779, AMD3100 (kind gifts from José Esté) or anti-hCCR5 mAb 2D7 (BD Pharmingen, San Diego, CA) for 15–30 min when indicated. Subsequently, cells were challenged with HIV-1_YU-2 _BlaM-Vpr virions (50 ng p24 CA per 2 × 10^6 ^T-cells) for 3–4 h, washed, and then loaded with CCF2/AM dye overnight. Fusion was monitored with a three-laser BD FACSAria Cell Sorting System (Becton Dickinson, San Jose, CA).

### Quantification of HIV-1 2-LTR circles and total HIV-1 cDNA

The relative amounts of 2-LTR circles and/or total HIV-1 cDNA in extracts from *ex vivo*-infected T-cell cultures or *in vivo*-infected splenocytes were determined by real-time PCR with the ABI 7500 sequence detection system (Applied Biosystems, Foster City, CA) essentially as described [22]. Results for HIV-1 cDNA species were normalized to the amount of cellular DNA, which was quantified in a parallel amplification of rat GAPDH gene DNA or human RNaseP gene DNA (Applied Biosystems). Genomic standards were derived from dilutions of genomic DNA extracted from uninfected primary rat or human T-cell cultures and uninfected Rat2 or HeLa cells. The lowest limit of detection was 0.001 2-LTR circle copies per ng DNA and 0.02 total HIV-1 cDNA copies per ng DNA. All samples were run in duplicate or triplicate. Data analysis was performed using the 7500 System Software (Applied Biosystems).

### Quantification of integrated HIV-1 DNA

The procedure used to establish the quantitative nested PCR strategy for HIV-1 integrants in the rat genome and the generation of cell lines that served as integration standards, Rat2^int ^and HeLa^int^, is described in the Results section and in Fig. 4. Briefly, in a first PCR reaction, HIV-1 integrants were amplified by one primer annealing to the LTR (primer #1521 [30]), which contains a lambda-phage heel sequence at the 5' end, and by two outward-facing primers that target the highly redundant identifier (ID) consensus sequence within the rat BC1 RNA gene (primers #1734, 5'-GGTAACTGGCACACACAACC-3' and #1782, 5'-CTGAGCTAAATCCCCAACCC-3'). The conditions for the first PCR were (a) 3 min at 94°C, (b) 12 cycles of 30 sec at 94°C, 30 sec at 57°C, 4 min at 72°C, and (c) 10 min at 72°C. The second PCR amplified the first-round amplicon with a lambda-specific primer (primer #1522 [30]) and an LTR primer (#1523, 5'-TGACTAAAAGGGTCTGAGGGATCT-3') and an LTR-specific probe (probe #1524, 5'-(FAM)-TTACCAGAGTCACACAACAGACGGGCA-(rhodamine (TAMRA)-3'). The second-round cycling conditions were identical to those used to determine the total amounts of HIV-1 cDNA and 2-LTR circles [22]. For the PCR to detect HIV-1 integrants in human cells, the conditions and reagents were identical, except that a primer pair targeting the *Alu *elements served as cellular anchor primers (forward primer #1719 [30] and reverse primer #1720 [54]). For each integration PCR, a control reaction in which the cellular primer pair was omitted was run in parallel. This value was routinely subtracted from the total signal. For standard curves, dilutions of genomic DNA from Rat2^int ^and HeLa^int ^covering 3.9 logs were used. The lowest detection standard of the PCR was 0.07 and 0.05 HIV-1 integrants per ng DNA, for integrated provirus in rat and human genomic DNA, respectively.

### In vivo-infections in transgenic rats

Three hCD4/hCCR5-transgenic rats (age: 11 weeks; weights: 196–222 g) were anesthetized and challenged by tail vein injection via a plastic catheter with HIV-1_YU-2 _(corresponding to 3000 ng p24 CA per rat), essentially as described [11,22]. One hCD4-single-transgenic rat, inoculated with the identical virus dose, served as negative control. On day 4 post-challenge, all animals were sacrificed with CO_2 _and bilateral thoracotomy, and spleens were removed. Total DNA was prepared from single-cell suspensions of splenocytes with DNeasy tissue kits (Qiagen, Hilden, Germany) and analyzed by real-time PCR.

### HIV-1 single-round infections

Activated primary T-cells and T-cell lines (3 × 10^6^), adherent cell lines (0.8–1 × 10^5^), or primary macrophages (~2 × 10^5 ^cells) from rats and humans were pretreated with efavirenz (10 μM, 1 h, Bristol-Myers Squibb, Uxbridge, UK) where indicated and subsequently challenged with single-round HIV-1_NL4-3_E^- ^GFP reporter viruses pseudotyped with the indicated Envs (corresponding to 50–200 ng HIV-1 p24 CA). On day 3 after infection, the percentage of GFP-positive cells and the GFP mean fluorescence intensity (MFI(GFP)) were determined on a FACSCalibur using BD CellQuest Pro 4.0.2 Software (BD Pharmingen).

### Nucleofection of primary rat T cells

Activated primary rat T cells were transfected by nucleofection as described [35]. For the analysis of the effect of transiently expressed human Cyclin T1, the expression vector phCyclin T1 [15] was utilized. Flow cytometric analysis of viable, GFP-positive cells was performed 20 hours post nucleofection as reported.

## Competing interests

The author(s) declare that they have no competing interests.

## Authors' contributions

CG, NM, HGK, WCG, and OTK designed the study. CG conducted the majority of the experiments. NM performed provirus/CyclinT1 cotransfection studies in primary T-cells. IA generated virus stocks and primary cell cultures and performed and analyzed infection experiments. VH cloned the MoMLV-GFP construct and characterized the recombinant virus. HMT conducted and analyzed the mouse T-cell infection experiment. HGK assisted CG and OTK with data interpretation. CG and OTK wrote the paper. All authors approved the final manuscript.
